# The current studies on the mechanisms of lactic acid bacteria as probiotics in the alleviate of bovine diseases: a review

**DOI:** 10.3389/fcimb.2026.1899278

**Published:** 2026-08-18

**Authors:** Chun-Ling Guo, Lin-Yue Zhou, Shun-Yi Ji, Han-Jia Zhang, Zhi-Rui Ren, Xiao Tang, Hao Dong, Qing-Feng Meng

**Affiliations:** 1 College of Life Sciences, Jilin Agricultural University, Changchun, Jilin, China; 2 Engineering Research Center of Bioreactor and Pharmaceutical Development, Jilin Agricultural University, Changchun, China

**Keywords:** bovine diseases, gut microbiota, immunoregulation, lactic acid bacteria (LAB), mucosal barrier, probiotics

## Abstract

Lactic acid bacteria (LAB) are an encouraging probiotic option to antibiotics in treating typical bovine diseases, including but not limited to, mastitis, inflammatory bowel disease, diarrhea, and endometritis. The review discusses the therapeutic mechanisms of LAB in a systematic manner including competitive exclusion of pathogens, synthesis of antimicrobial products such as organic acids, bacteriocins, and short-chain fatty acids, recovery of mucosal barrier integrity by regulating tight junctions, and suppression of pro-inflammatory pathways, such as NF-κB and MAPK, to modulate the host immune response. All these mechanisms lower the inflammation, improve the condition of the gut and mammary glands and increase the level of productivity. LAB are an effective and long-term solution to decrease the consumption of antibiotics and antibiotic resistance. This review will form a theoretical basis of introducing LAB into veterinary practice and promote sustainable animal husbandry.

## 1 Introduction

Cattle are a vital type of livestock globally, providing humans with multifaceted practical value (Durana et al., 2023). Not only do they represent an important source of high-quality meat and dairy products but also one of the fundamental foundations of the agricultural economy, with a substantial effect on the sustainable development of agriculture. Nonetheless, cattle health is a direct threat to these values. Diseases of common bovines, such as mastitis, colitis, diarrhea, and ruminal acidosis are prevalent, highly common, and very destructive diseases in the world population of livestock (Autio et al., 2021).

The causation of reduced milk yield and poor milk quality among dairy cows due to the bovine mastitis, mainly caused by pathogenic microbial infection. Based on statistics calculated by using the data in FAO database, the total economic losses of cattle diseases over the past few years have been estimated at around 12 billion US dollars, making a significant impact on the livelihoods of farmers and other associated industries around the globe (Chen et al., 2021). Young animals, especially calves, often suffer from high mortality rate and growth retardation caused by bovine colitis and diarrhoeal diseases (e.g. *Escherichia coli* and *viral diarrhoea*), which directly affects the reproductive efficiency and seriously endangers the sustainable development of the livestock industry (Çömlekcioğlu et al., 2024). There is also another extremely important problem that should not be ignored the condition known as ruminal acidosis, which is typically triggered by high-concentrate feeding, resulting in digestive and metabolic disorders, lower milk fat percentage, and ultimately, the poor quality and competitive capabilities of dairy products (Hindman, 2023). Besides being very costly to treat, these diseases also increase the risks of breeding. Even worse, the misuse of antibiotics has led to the increase in the development of antimicrobial resistance, which can not only threaten food safety and human health but also cause unforeseeable possible threats to human health (Bayne et al., 2025).

Being one of the most promising therapeutic strategies with high potential, LAB have demonstrated high value of application in many areas. LAB can maintain the intestinal microbial balance and provide an inhospitable environment to the proliferation of pathogenic bacteria through the production of organic acids, bacteriocins and other metabolites, which may be used as a preventive and therapeutic measure against intestinal diseases (Zapaśnik et al., 2022). They also show a tremendous immunostimulatory activity, inducing the immune response of the host and enhancing the resistance to diseases. The regulation of metabolic parameters as blood glucose and blood lipids by LAB is a useful function towards the prevention and treatment of metabolic diseases, which promotes good health in cattle (He et al., 2020).

Additionally, LAB have been applied in the food and pharmaceutical industries with increasing frequency. They may be applied to create functional foods, which means that they provide customers with healthier and more nutritious options. Probiotic formulations of LAB can also be developed to treat bowel diseases and regulate the immune system. Recent experiments have largely involved human or mouse models but there have been similar studies in other animals. Such applications indicate the wide opportunities and feasibility of LAB as well as help to enhance the condition of the population and facilitate the growth of biotechnology (Ayed et al., 2024).

This review points to the benefits of LAB as the replacement of antibiotics with high safety, no risk of developing drug resistance, and no residual pollution that is in line with the idea of sustainable development. It gives theoretical justification of the practical implementation of LAB in veterinary facilities and animal husbandry and presents new approaches to solving global issues in livestock farming in terms of disease prevention, financial gains, and environmental security.

However, the clinical use of LAB is constrained by several unresolved issues. Currently, there is a pronounced scarcity of direct *in vivo* evidence, particularly from large-scale, multi-center field clinical trials in cattle, with most mechanistic insights still heavily relying on murine models or *in vitro* assays that lack anatomical and immunological equivalence to ruminants. Furthermore, therapeutic efficacy remains highly strain-specific, and standardized guidelines for optimal dosing, target-site delivery, and formulation technologies are virtually non-existent. Future research must also prioritize assessing the long-term ecological impacts of exogenous LAB on host endogenous microbiota, evaluating the safety risks of antibiotic resistance gene transfer, and performing comprehensive cost-benefit analyses to validate the economic viability of LAB-based interventions in herd health management.

## 2 Methods: literature search and selection strategy

A systematic literature search was performed in four electronic databases, namely PubMed, Web of Science, ScienceDirect, and China National Knowledge Infrastructure (CNKI), covering the period from January 2000 to July 2026. The search strategy combined controlled vocabulary and free-text keywords related to the following concepts: lactic acid bacteria (e.g., “*lactic acid bacteria*”, “*Lactobacillus*”, “probiotics”), bovine inflammatory diseases (e.g., “bovine mastitis”, “bovine diarrhea”, “bovine endometritis”), and protective mechanisms (e.g., “protective mechanism”, “action pathway”, “immune regulation”). Only peer-reviewed original research articles published in English or Chinese were considered.

The screening process followed a structured, stepwise approach. The initial search yielded 2,683 records. After removing 966 duplicates, 1,717 records remained for title and abstract screening. Of these, 1,028 were excluded based on irrelevance to the review question, and an additional 419 were removed owing to inappropriate publication types. The remaining 270 full-text articles were assessed against predefined eligibility criteria. During this phase, 204 articles were excluded for the following reasons: studies not using lactic acid bacteria as the intervention or lacking clear strain information (n=42), studies not involving immunomodulatory or molecular pathway mechanisms of LAB (n=106), studies investigating diseases other than the three target bovine inflammatory conditions (n=29), and studies with purely *in vitro* designs without *in vivo* data (n=27). Ultimately, 66 studies met all inclusion criteria and were included in the final synthesis.

Given the limited availability of *in vivo* mechanistic studies directly conducted in cattle due to high costs, long experimental cycles, and significant individual variability, the present review additionally incorporated murine model studies to comprehensively elucidate the conserved immunomodulatory and molecular signaling pathways underlying the protective effects of LAB against bovine inflammatory diseases.

## 3 Basic characteristics of lactic acid bacteria

### 3.1 Classification and distribution of lactic acid bacteria

Lactic acid bacteria constitute a group of Gram-positive bacteria that synthesise lactic acid through fermentation of sugars. They are widely distributed in humans, animals, plants, and fermented foods; most can survive in hypoxic or anaerobic environments and are characterised by their Gram-positive nature, low GC content, acid tolerance, and non-spore formation (Wang et al., 2021). LAB primarily belong to the genera *Acetilactobacillus*, *Lacticaseibacillus*, *Lactiplantibacillus*, *Leuconostoc*, *Limosilactobacillus*, and *Lactococcus* (Zheng et al., 2020). Their distribution in nature is extremely broad, and they are almost ubiquitous. In the animal gastrointestinal tract, LAB are key members of the microbial flora. In conjunction with other microorganisms, they constitute a complex intestinal microecosystem and play a vital role in maintaining intestinal microecological balance. They support host health through various mechanisms, including the production of beneficial metabolites and the regulation of immune responses (Jia et al., 2025). The LAB are essential in any dairy product manufacturing process. As a case study of fermented dairy products, LAB can break down the lactose and release organic acids during the fermentation process, which is not only useful to give dairy products special taste and flavor but also adds to their nutritional value and health-promoting properties (Ziarno et al., 2023).

### 3.2 Distribution and effects of lactic acid bacteria in cattle

Lactic acid bacteria associated with cattle predominantly encompass the genera *Lactobacillus*, *Lactococcus*, and *Bifidobacterium*. Among these, *Lactiplantibacillus plantarum*, *Lacticaseibacillus casei*, *Limosilactobacillus reuteri*, and *Ligilactobacillus salivarius* represent the most extensively investigated and applied species. These bacterial communities widely colonize the bovine gastrointestinal tract and mammary glands. Within the gastrointestinal tract, although LAB are not the absolute dominant taxa in the rumen of adult ruminants, they play a crucial role in the digestive tract of newborn and pre-weaning calves. As animals age and dietary structures shift, LAB successfully colonize the rumen, small intestine, and cecum, playing a pivotal role in maintaining intestinal epithelial barrier integrity and microecological homeostasis (Addis et al., 2016).

Conventionally, the mammary glands of healthy cows were perceived to be sterile. However, recent high-throughput sequencing technologies have confirmed the residency of distinct microbial communities within both the internal mammary tissues and milk of healthy cattle. *Lactococcus lactis, Lactiplantibacillus plantarum*, and certain *Lactobacillus species* are common residents in healthy bovine milk. These LAB establish colonization via the “gut-mammary axis” or through retrograde influx via the teat canal. Consequently, they interact with the host mammary immune system and serve as a natural biological barrier against the invasion of mastitis pathogens (Liu et al., 2023).

### 3.3 Effects of lactic acid bacteria in cattle

Lactic acid bacteria exclude the colonization of pathogens (such as *Escherichia coli and Salmonella*) by competitively binding to intestinal epithelial cell receptors. In adult dairy or beef cattle, specific LAB strains can synergistically stabilize ruminal pH, thereby preventing subacute ruminal acidosis (SARA) induced by high-concentrate diets (Li et al., 2026). Furthermore, LAB colonizing the mammary gland or migrating to it via oral administration can stimulate local mammary immune cells, promoting the secretion of immunoglobulins. During mastitis caused by *Staphylococcus aureus* or *Streptococcus uberis*, beneficial LAB can downregulate the expression of pro-inflammatory cytokines, thereby mitigating inflammatory injury to the mammary tissue (Fukuyama et al., 2020).

### 3.4 Effects of lactic acid bacteria on adult cattle versus calves

The regulatory effects of probiotics in cattle exhibit a profound age-dependent sensitivity. At birth, the gastrointestinal tract of neonatal calves is virtually sterile, and anatomically, the rumen remains underdeveloped, leaving digestion to rely predominantly on the abomasum. During this critical window, the administration of lactic acid bacteria can rapidly occupy ecological niches, thereby promoting early intestinal epithelial barrier development and stimulating the establishment of a multi-tiered immune system (Hawkins et al., 2024). In contrast, adult cattle possess a fully functional and extraordinarily complex rumen microbial barrier. Exogenous lactic acid bacteria entering the adult gastrointestinal tract must not only navigate surveillance by the host’s mature immune system but also withstand fierce exclusion by the massive indigenous rumen microbiota. Consequently, the response to probiotic supplementation in adult cattle is typically less pronounced than in young calves; its therapeutic efficacy is largely confined to stabilizing ruminal pH or mediating transient systemic immunomodulation, rather than achieving long-term microbiota reconstruction (Duet al., 2023).

### 3.5 Passive immune transfer and immunotolerance

Neonatal calves absorb immunoglobulins (IgG) from colostrum via non-specific macromolecular transcytosis within the first 24 hours postpartum. During this transient window, although maternal immune factors in colostrum possess inherent antimicrobial properties, they exhibit higher immunotolerance toward homologous probiotics. Administering multi-strain probiotics prior to the first colostrum feeding, or co-administrating them with colostrum, significantly elevates serum IgG concentrations in calves. This synergism indicates that probiotics can optimize the passive transfer of colostral macromolecules (Shams et al., 2022).

## 4 Mechanisms of lactic acid bacteria in the treatment of bovine mastitis

### 4.1 Overview of the etiology and pathogenesis of mastitis

**Figure 1:**
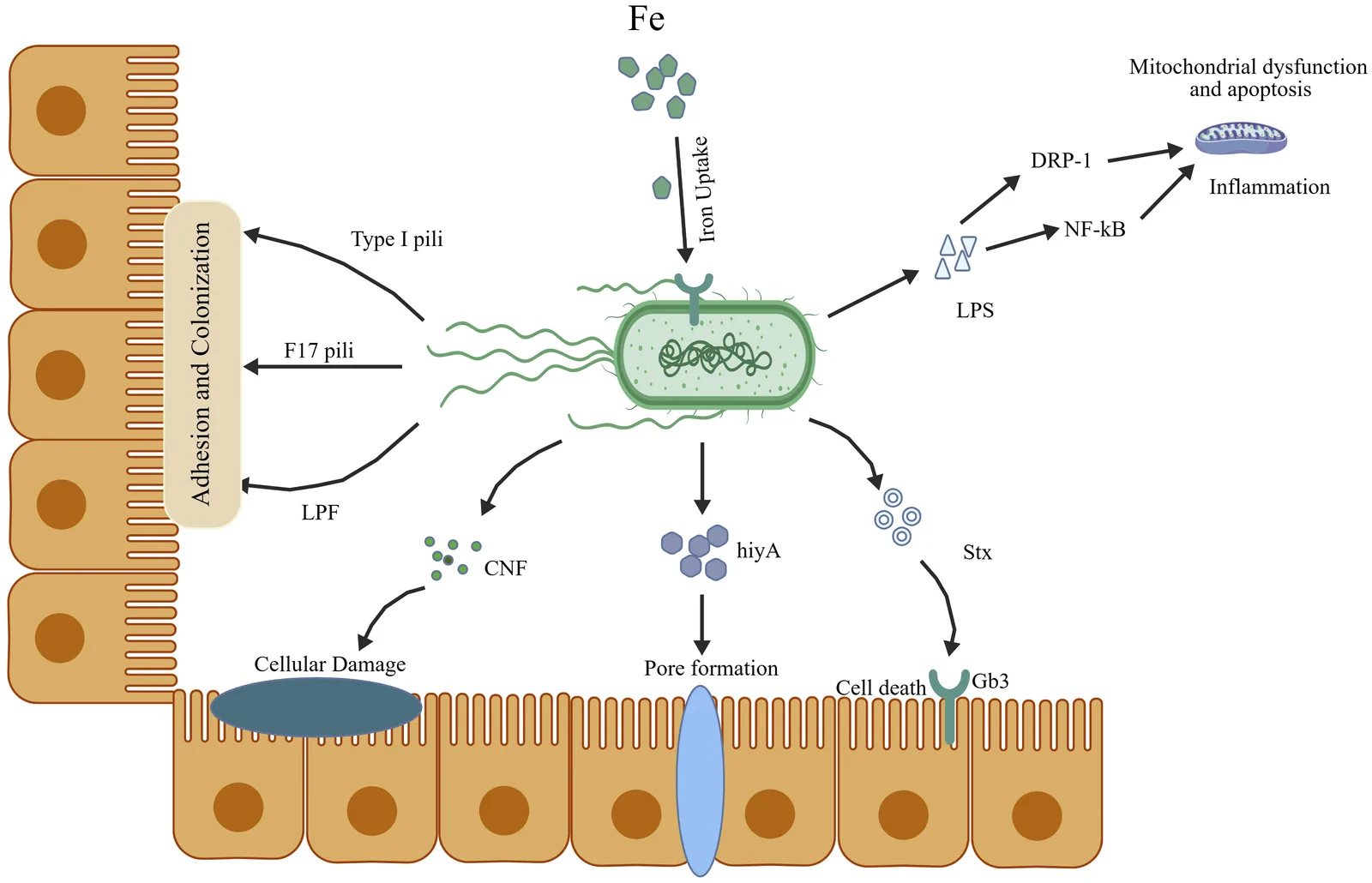
Schematic representation of the roles of *Escherichia coli* virulence factors in the pathogenesis of mastitis. *E. coli* recognizes and binds to host epithelial cells via multiple fimbriae: F17 fimbriae target N-acetyl-D-glucosamine receptors, type 1 fimbriae bind to mannose residues on cell surfaces through apical adhesins, and long polar fimbriae (LPF) adhere to sialic acid-containing N-acetyllactosamine structures. Type I pili (fimH), F17 pili (f17A), and other fimbrial structures mediate specific adhesion to mammary epithelial cells. The synergistic action of these fimbriae allows bacteria to firmly attach to host epithelial cells and achieve colonisation. *E. coli* utilises host iron carriers, such as transferrin, and acquires iron—an essential nutrient for bacterial growth and proliferation—via the iron uptake system, enabling survival and massive proliferation in iron-deficient host environments. In terms of virulence and cellular injury, lipopolysaccharide, the major pro-inflammatory component located in the bacterial outer membrane, consists of lipid A, core oligosaccharide, and O-antigen repeating units. α- and β-hemolysin (hlyA) form pores in host cell membranes and directly induce cell death. Cytotoxic necrotising factor (CNF) directly damages cells and elicits intense inflammation. Shiga toxin (Stx) binds to the cell surface receptor Gb3 and directly triggers apoptosis. In addition, LPS activates the DRP-1 and NF-κB signalling pathways, causing mitochondrial dysfunction and inflammatory responses, which ultimately mediate apoptosis. Through these mechanisms, *E. coli* achieves comprehensive destruction of host cells and establishes pathogenic infections.

Bovine mastitis is an inflammatory condition affecting the mammary gland, resulting in pathological changes in glandular tissue and abnormal milk, which causes substantial economic losses to the global dairy industry. As the leading infectious disease contributing to economic losses in the global dairy industry, it results in direct economic losses exceeding 35 billion US dollars annually and indirectly raises the production costs of dairy products and directly increases dairy prices. Antibiotic therapy is commonly employed in the treatment of clinical mastitis; however, the excessive use of such treatments has led to the development of antibiotic resistance and, in some cases, multidrug resistance among major pathogenic bacteria (Catalano et al., 2022), which constitutes a significant threat to human health and compromises the effectiveness of mastitis treatment (Alhaji et al., 2019). *Staphylococcus* and *Escherichia coli* are the main pathogenic bacteria causing bovine mastitis (Srithanasuwan et al., 2024). Among them, the pathogenesis of *Escherichia coli* involves *lipopolysaccharide* as its key virulence factor. LPS activates the TLR4 signalling pathway via the CD14/TLR4/MD2 complex, triggering both MyD88-dependent and TRIF-dependent signalling pathways. Activation of the NF-κB and MAPK pathways leads to the release of pro-inflammatory cytokines, such as TNF-α, IL-6, and IL-1β. Through binding to TLR4, neutrophil infiltration induces an oxidative burst, resulting in an increased apoptosis rate of acinar epithelial cells. After endotoxins enter the bloodstream, they induce systemic inflammatory response syndrome, cause endotoxic shock, and elicit a strong immune response (**Figure 1**) (Zhuang et al., 2023).

**Figure 2:**
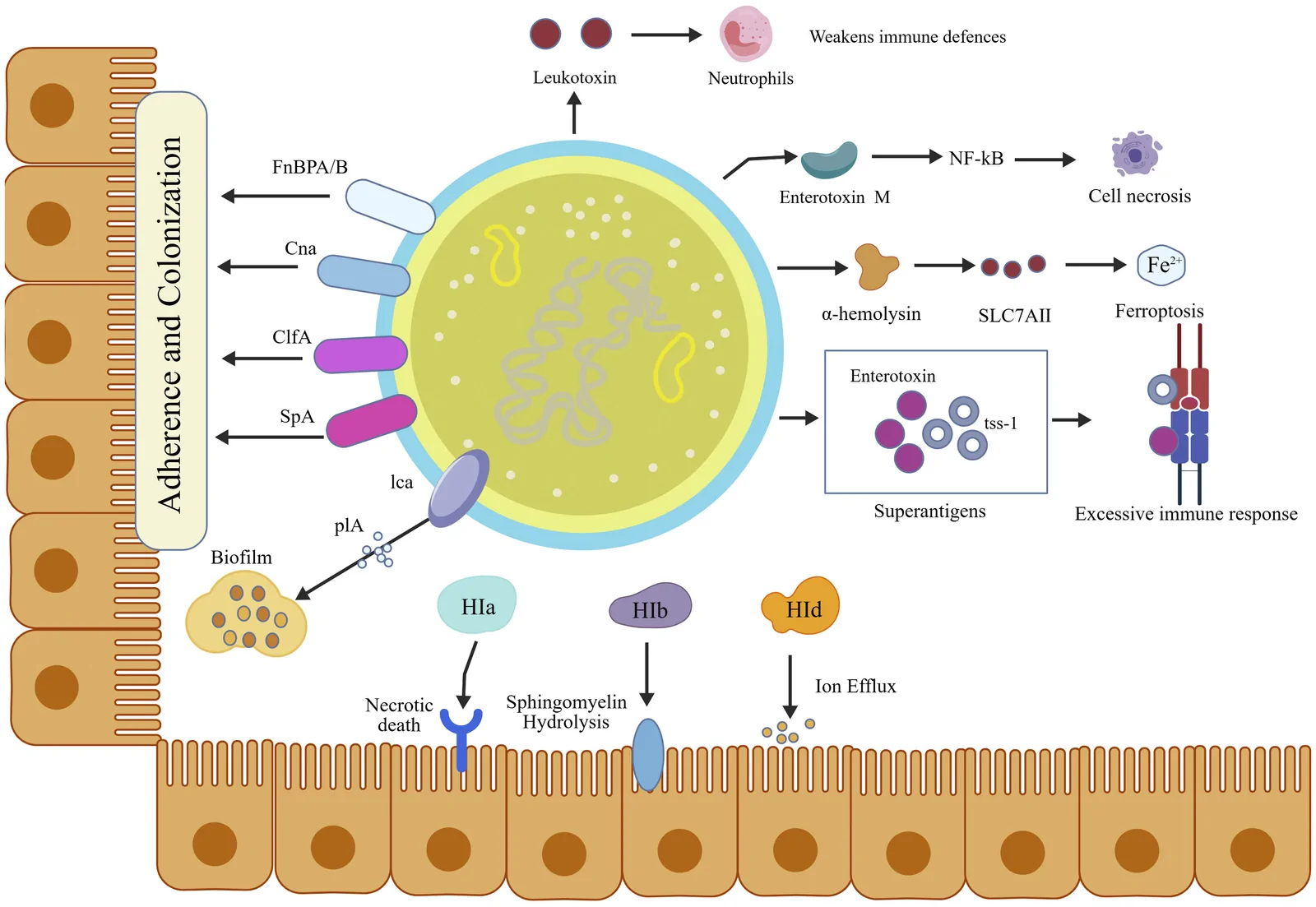
Illustrative representation of the involvement of *Staphylococcus aureus* virulence factors in the development of mastitis. *S. aureus* adheres to the host extracellular matrix and initiates colonisation via surface proteins: FnBPA/B binds fibronectin, Cna binds collagen, and ClfA binds fibrinogen, while SpA binds immunoglobulins and interferes with antibody function, thereby completing the initial stage of infection. In addition, *S. aureus* regulates exopolysaccharide synthesis in combination with ica and plA, forming a three-dimensional biofilm structure that enhances bacterial resistance to antibiotics and host immunity, leading to chronic persistent infection that is difficult to eliminate. *S. aureus* secretes multiple toxic factors. Among membrane-damaging toxins, α-hemolysin (Hla/hla) forms pores in the cell membrane and lyses cells; β-hemolysin (Hlb) increases membrane permeability and hydrolyzes sphingomyelin to disrupt membrane integrity; and δ-hemolysin (Hld) induces ion efflux and disturbs cellular homeostasis. Leukotoxins specifically lyse immune cells, such as neutrophils, to weaken host defense. Superantigens, including enterotoxins and toxic shock syndrome toxin (tsst-1), non-specifically activate a large number of T cells, triggering a cytokine storm and simultaneously activating the NF-κB pathway to induce apoptosis, ferroptosis, and necrosis. Among these, superantigens and membrane-damaging toxins are key factors responsible for severe clinical conditions, such as sepsis and toxic shock, induced by *S. aureus*.

The pathogenicity of *Staphylococcus aureus* involves adhesion to host cells and the extracellular matrix via surface proteins and biofilm formation, which facilitates colonisation and resistance against host defences. It also regulates the expression of virulence factors through the *Agr* quorum-sensing system (Mao et al., 2023). Massive expression of virulence factors damages bovine tissues, allowing bacteria to invade deeper host tissues. More seriously, *S. aureus* produces substances such as superantigen toxins, protein A, and polysaccharide capsules, which help bacteria evade recognition and attack by the immune system, ultimately leading to infection and mastitis in dairy cows, severely affecting milk production performance and the quality and safety of dairy products (**Figure 2**) (Field et al., 2021).

### 4.2 Inhibitory mechanisms of lactic acid bacteria against mastitis pathogens

#### 4.2.1 Regulate the mammary microenvironment

Lactic acid bacteria can produce antibacterial substances to inhibit the proliferation of pathogenic bacteria. Antibacterial compounds such as acidocin secreted by lactic acid bacteria can directly destroy the membrane integrity of pathogenic bacteria, leading to decreased membrane potential and increased intracellular K^+^ leakage. By inhibiting ribosomal protein synthesis, acidocin reduces ATPase activity, this process leads to the efflux of vital cellular constituents and the suppression of bacterial proliferation. Lactic acid bacteria possess auto-aggregation capabilities and surface hydrophobicity, allowing them to competitively bind to mammary epithelial cells by secreting adhesin-like components. This significantly reduces the adhesion and internalization of *Staphylococcus aureus* in bovine mammary epithelial cells (bMECs) (Bouchard et al., 2013). In *in vitro* studies, pretreatment of bMECs with *Lactobacillus casei BL23* markedly downregulated the expression of pro-inflammatory cytokines stimulated by *S. aureus*, thereby buffering excessive immune responses (Souza et al., 2018). The short-chain fatty acids (SCFAs), mainly acetate, propionate, butyrate and caproate play both the role of the pathogen inhibitor and barrier repair. Sodium propionate, a critical component of the blood-milk barrier, facilitates the repair of LPS-induced damage to this barrier and modulates the expression of tight junction proteins and histone deacetylase (HDAC) (Wang et al., 2017).

#### 4.2.2 Repair of mammary gland barrier function

Lactic acid bacteria play a key role in alleviating mastitis by repairing the mammary gland barrier, with core mechanisms involving the synergistic effects of tight-junction protein regulation and epithelial regeneration. *Escherichia coli* infection reduces the expression levels of tight-junction proteins claudin-3 and occludin in mammary epithelial cells and causes protein mislocalization (Li et al., 2023). Treatment with lactic acid bacteria reverses this damage by activating the PI3K/Akt signalling pathway, upregulating claudin-3 gene expression, increasing occludin protein synthesis, inhibiting pathogen-induced *MLCK* kinase activity, preventing myosin light chain phosphorylation, and maintaining cytoskeletal stability (Qiu et al., 2022). Using an *in vivo* mouse model, Huang et al. confirmed that Nisin Z significantly reduced inflammatory cell infiltration in mammary tissues during lipopolysaccharide induced mastitis. Mechanistically, Nisin Z specifically inhibited the MAPK signaling pathway—as evidenced by the suppressed phosphorylation of *ERK1/2* and *p38*—thereby decreasing the release of TNF-α, IL-1β, and IL-6 while elevating the expression of the anti-inflammatory cytokine *IL-10*, which ultimately enhanced the integrity of the blood-milk barrier (Huang et al., 2022). Furthermore, by integrating an *in vitro* bMECs model with an *in vivo* lactating mouse model, a subsequent study revealed that *Lactobacillus casei* Zhang effectively attenuated pathogen-induced desmosome damage. The core mechanism involves the profound upregulation of both the mRNA and protein expression of tight junction proteins, which successfully reversed the pathogen-induced increase in blood-milk barrier permeability (Zheng et al., 2021).

### 4.3 Limitations and future perspectives

#### 4.3.1 Current limitations

At present, the vast majority of *in vivo* mechanistic studies heavily rely on murine mastitis models, whereas mechanistic investigations directly utilizing dairy cows as *in vivo* subjects remain exceedingly scarce. Dairy cows possess a well-developed teat canal and teat sphincter, which serve as the primary physical defense barrier against pathogen invasion—anatomical structures that are absent or poorly correlated in scale within murine models. However, the purchase and husbandry of lactating dairy cows, alongside the construction of dedicated experimental farms meeting stringent biosafety standards, require prohibitively high financial investments, which primary drives the widespread use of mice as alternative experimental animals. Furthermore, performing intramammary infusions of *Escherichia coli* or *Staphylococcus aureus* in live cows induces severe pain, systemic acidosis, and even mortality. Consequently, these procedures face extremely rigorous ethical reviews and carry a high risk of animal culling.

#### 4.3.2 Future directions

Priority should be given to promoting the application of bovine mammary organoids and mammary-on-a-chip technologies. These advanced platforms can highly recapitulate the native three-dimensional architecture, blood-milk barrier permeability, and fluid dynamics of the bovine mammary gland, thereby providing *in vitro* data of greater clinical relevance without compromising animal welfare. Future research should leverage multi-omics approaches (including metagenomics, transcriptomics, and metabolomics) to further elucidate the epigenetic regulations and signaling molecular mechanisms through which lactic acid bacteria modulate host immunity and fortify the mammary barrier.

## 5 Mechanisms of lactic acid bacteria in the management of bovine diarrhea

### 5.1 Overview of the etiology and pathogenesis of diarrhea

Calf diarrhea is a prevalent condition among young animals and constitutes the primary cause of mortality and economic losses in pre-weaned calves, imposing heavy economic burdens on the livestock industry. This complex disease results from co-infection with multiple pathogens, including viruses, bacteria, parasites, and others, and its pathogenesis is also strongly influenced by environmental and management factors.

Common infectious pathogens include bovine rotavirus (BRV), bovine viral diarrhea virus (BVDV), bovine coronavirus (BCoV), enterotoxigenic Escherichia coli (*ETEC*), and *Cryptosporidium parvum* (Xie et al., 2023). Bovine rotavirus (BRV) causes diarrhea and intestinal cell damage in calves. After entering the host, BRV binds to the host intestinal epithelial ganglioside *GM1* receptor via the viral surface protein *VP4*, targeting and infecting mature epithelial cells at the tips of small intestinal villi. Loss of mature enterocytes leads to nutrient depletion and subsequent metabolic acidosis. In neonatal calves, reduced intestinal lactase activity exacerbates osmotic dysregulation following milk intake. Shortened villus height reduces the absorptive surface area, and undigested lactose accumulates in the intestinal lumen, triggering osmotic imbalance and reverse fluid flux (Veshkini et al., 2024). Continued milk feeding creates an osmotic vicious cycle, sharply increasing fecal water content and resulting in typical watery diarrhea. After infecting calves, *Cryptosporidium parvum* alters the intestinal mucosal transcriptome. The secreted *CpABC* transporter competitively inhibits the host sodium glucose cotransporter, reducing glucose absorption. Concurrently, infection downregulates the expression of E-cadherin and disrupts the continuity of tight junction proteins claudin-3 and occludin, increasing intestinal permeability. It also interferes with the expression of genes related to ABC, SLC transporters, and ion channels, impairing transmembrane nutrient transport, acid-base balance, and osmotic homeostasis. These disturbances lead to abnormal nutrient absorption, metabolic disorders, and clinical manifestations including diarrhea and metabolic acidosis (Kumar et al., 2018).

### 5.2 Mechanisms of lactic acid bacteria in the prevention and treatment of diarrhea

#### 5.2.1 Regulation of intestinal microecology

Lactic acid bacteria establish an efficient antibacterial defense system through a triple mechanism: colonisation resistance, chemical inhibition, and biofilm disruption, which exert inhibitory effects against *Escherichia coli*, *Staphylococcus aureus*, and *Salmonella Typhimurium* (Gómez et al., 2016). The adhesive capacity of lactic acid bacteria, evaluated using *in vitro* cell adhesion assays, demonstrates that they can occupy more adhesion sites in the intestine, compete with pathogenic bacteria, block pathogen colonisation, and occupy intestinal niches, thereby making it difficult for pathogens to establish infection and reducing the risk of disease. Lactic acid bacteria produce a variety of antibacterial substances. For example, *Lactobacillus reuteri DSM 17938* secretes reuterin, a potent antimicrobial compound. Reuterin drastically downregulates the expression of *hilA*, a key gene encoding the type III secretion system in Salmonella, and reduces pathogen ATP synthesis rate by inducing reactive oxygen species burst and disrupting membrane potential (Yoo et al., 2023).

Reuterin also inhibits *Staphylococcus aureus* biofilms by degrading the exopolysaccharide matrix. Combined with the acidic environment generated by lactic acid, it markedly reduces the number of viable bacteria within biofilms. Reuterin effectively inhibits the proliferation of *E. coli*, *Salmonella*, *Clostridium difficile*, *Campylobacter jejuni*, and other pathogenic microorganisms, thereby playing a crucial role in the prevention and treatment of diarrhoea (Pramudito et al., 2024). Zhao et al. reported that *Lactobacillus plantarum* and *Lactobacillus reuteri* regulate intestinal flora. They mitigate intestinal inflammation by inhibiting pro-inflammatory factors such as IL-6 and TNF-α, and by obstructing the MyD88/NF-κB/p65 signaling pathway, significantly increase the relative abundance of lactobacilli, relieve diarrhea caused by *E. coli* infection, and elevate the level of beneficial intestinal bacteria (Zhao et al., 2025). Liu et al. demonstrated that compound probiotics significantly affect the intestinal flora of calves. The dominant phyla in the calf intestine became *Firmicutes*, *Actinobacteriota*, and *Bacteroidota*, with *Bifidobacterium* being the most abundant genus. The intestinal bacterial community in the compound probiotic group exhibited tighter clustering and a lower diarrhea rate (Liu et al., 2022).

#### 5.2.2 Improvement of intestinal digestion and absorption function

Cell surface hydrophobicity, auto-aggregation and co-aggregation properties of lactic acid bacteria have been linked to the functioning of the intestinal barrier. The hydrophobicity of *Lactobacillus rhamnosus* A012 is very good and it can also co-aggregate with *Escherichia coli* causing diarrhea to create a biological barrier and prevent the loss of integrity of the intestinal mucosa and provide normal digestion and absorption (Obisesan et al., 2024). In their report, Xu X et al. stated that *Lactobacillus reuteri* ZY15 significantly alters the composition of the intestinal microbiota, enhances the levels of *Akkermansia* and *Clostridium sensu stricto* UCG.014, restores the colonization resistance of the intestine, and increases the activity and absorption of the intestine (Xu et al., 2025). These results suggest that the lactic acid bacteria decrease the pathologic effects of the harmful bacteria on the intestinal mucosa, inhibit the ability of harmful bacteria to disturb the physiological absorption and excretion of intestinal water and electrolytes and stabilize the intestinal water-electrolyte balance (Zamojska et al., 2021).

#### 5.2.3 Field trials of lactic acid bacteria in mitigating diarrhea

Under practical farming conditions, the efficacy of lactic acid bacteria in reducing the incidence of calf diarrhea is robustly supported by extensive clinical data. In a large-scale, randomized controlled field trial involving multiple commercial dairy farms, calves orally administered a multi-strain probiotic complex (*comprising Lactobacillus* spp*, Bifidobacterium* spp., and *Enterococcus faecium*) daily for the first 28 days of life exhibited a significantly lower incidence of diarrhea. Furthermore, the proportion of calves requiring antibiotic therapy for diarrheal illness was markedly lower than that in the control group (Wu et al., 2021). A meta-analysis synthesizing data from dozens of calf field trials globally revealed that colonization with specific LAB strains reduced the relative risk (RR) of pre-weaning calf diarrhea. However, the study simultaneously highlighted that the efficacy of these probiotics is heavily constrained by on-farm colostrum management practices, environmental hygiene, and the viable count (colony-forming units, CFU) of the probiotics administered (Signorini et al., 2012).

## 6 The mechanisms of lactic acid bacteria in the therapy of bovine endometritis

### 6.1 Overview of the etiology and pathogenesis of bovine endometritis

Based on clinical signs and diagnostic methods, bovine endometritis is mainly classified into two types: clinical endometritis and subclinical endometritis. Clinical endometritis is a local inflammation of the endometrium characterized by the presence of visible purulent uterine discharge. In contrast, subclinical endometritis lacks obvious clinical signs and cannot be observed directly with the naked eye; it is mainly diagnosed by endometrial cytology (Krasniqi et al., 2024). Postpartum dairy cows are susceptible to various factors, such as birth canal trauma, placental separation, immunosuppression, and shifts in uterine microbiota, which may lead to inflammatory diseases of the reproductive tract. The bovine uterus is frequently colonized by microorganisms, among which Escherichia coli and Trueperella pyogenes are the key pathogens (Becker et al., 2023). *Escherichia coli* often acts as a pioneer pathogen that damages the endometrial barrier and suppresses local immunity. Pyolysin produced by *Trueperella pyogenes* injures the endometrium and interacts synergistically or antagonistically with other bacteria (Dahiya et al., 2018).

### 6.2 Regulatory mechanisms of lactic acid bacteria against bovine endometritis

#### 6.2.1 Competitive exclusion

Lactic acid bacteria possess the capacity to inhibit the colonization of pathogens through their abilities of auto-aggregation and co-aggregation. Auto-aggregation is associated with biofilm formation and adhesion to host cells, whereas co-aggregation prevents pathogen colonisation (Liu et al., 2022). Different lactic acid bacteria exhibit distinct aggregation abilities and varying co-aggregation capacities against *Escherichia coli*, *Staphylococcus aureus*, *Salmonella Typhimurium*, and other pathogens. *Lactobacillus plantarum* and *Lacticaseibacillus camelliae* show strong co-aggregation with *E. coli*, which helps form a protective barrier and inhibits pathogen colonisation in the endometrium (Shafique et al., 2021). Many yak-derived lactic acid bacteria show strong probiotic potential by inhibiting pathogens, adhering to host cells, and surviving the gastrointestinal tract. These traits provide a solid theoretical basis for developing yak-origin microecological preparations (Zhang et al., 2022).

#### 6.2.2 Immunoregulation

*Lacticaseibacillus rhamnosus* GR-1 exhibits anti-inflammatory activity in bovine endometrial epithelial cells (BEECs). *E. coli* infection upregulates the mRNA levels of pro-inflammatory cytokines IL-6, *IL-8* and IL-1β, while pretreatment with GR-1 markedly reverses this elevation (Liu et al., 2016). Pathogen recognition receptors *TLR2*, TLR4, NOD1 and NOD2 are overexpressed upon *E. coli* challenge, which can be suppressed by GR-1 to block downstream inflammatory cascades. Mechanistically, GR-1 inhibits the phosphorylation of core proteins in both NF-κB and MAPK pathways, thereby restraining pathway activation and reducing the secretion of inflammatory mediators (Cao et al., 2024). Four lactobacilli strains including *L. rhamnosus*, *L. acidophilus*, *L. sakei* and *L. reuteri* alleviate *E. coli* infection of endometrial cells. Specifically, *L. acidophilus* secretes organic acids, hydrogen peroxide and antimicrobial peptides, and *L. sakei* produces specific bacteriocins to suppress pathogenic invasion (Li et al., 2024). Genís S et al. found that the combined treatment of *L. rhamnosus, Lactococcus lactis* and *L. reuteri* reduced the secretion of pro-inflammatory cytokines, and greatly relieved the pathological damage induced by *E. coli* in BEECs (Genís et al., 2017). Waehama et al. further reported that peptidoglycans derived from *L. rhamnosus* (PGN-Lr) and *L. acidophilus* (PGN-La) attenuate BEEC inflammation via modulating the *TLR2/1* signaling pathway (Waehama et al., 2025).

## 7 Future uses of lactic acid bacteria in bovine disease control

Natural probiotics, lactic acid bacteria, perform an extremely significant and unique function in the animal husbandry of today and the maintenance of human health. Lactic acid bacteria are non-toxic and safe to use in the host, so they are ideal substitutes to antibiotics (Kim et al., 2021). The excessive use of antibiotics has always been part of the conventional livestock production and medical care, which resulted in the growing severe antibiotic resistance, which is a significant concern to human health and livestock farming. Lactic acid bacteria act mostly through competitive exclusion. The pathogenic bacteria will try to colonize and cause diseases in the complex intestinal microecosystem whereas the lactic acid bacteria can preemptively attach to the intestinal adhesion sites and compete with pathogens to nutrients, thus effectively preventing the colonization of pathogens (Purevdorj et al., 2021).

By these mechanisms, the composition and amount of the intestinal microbiota are changed and the homeostasis of the microbiota is restored. Besides controlling gut microbiota, lactic acid bacteria play a key role in boosting host immunity (Mendes et al., 2021). They activate the intestinal mucosal immune system, induce the production of immunoglobulin A in gut related lymphoid tissue, the intestinal mucosal barrier and thus enhance the overall disease resistance of cattle. From a macroscopic viewpoint, not only does this decrease the rate of diseases and antibiotic use in cattle, but it also reduces the environmental pollution due to antibiotic residues, which is in line with the present view of green and sustainable development (Sylvere et al., 2023). However, there are still a number of obstacles in the practical use of lactic acid bacteria. On the positive side, lactic acid bacteria are highly specific to strains and the inhibitory effects against diseases vary greatly across strains. It is illustrated by the fact that some strains of *Lactobacillus plantarum* have good inhibitory effects against *Escherichia coli*-induced diarrhea, whereas others of L*. acidophilus* are more effective against Salmonella infection. This strain specificity complicates the selection of suitable lactic acid bacteria strains to apply to clinical therapy or livestock application (Yue et al., 2020). Conversely, the underlying mechanisms of the interaction between lactic acid bacteria and host immune systems are poorly understood. Although it is known that lactic acid bacteria are capable of stimulating the immune response, the exact signaling pathways and molecular processes involved are still unclear and need further research. The rumen environment in ruminants is another major issue to the implementation of lactic acid bacteria (Belo et al., 2021).

The rumen is described as having a low pH and a high concentration of volatile fatty acids, which can negatively impact the survival and colonization effectiveness of exogenous supplemented lactic acid bacteria. In these conditions, most lactic acid bacteria cannot survive and perform their beneficial roles, hindering the large-scale implementation of lactic acid bacteria in ruminant farming (Byanju et al., 2023). Lactic acid bacteria may be applied in conjunction with other therapeutic methods. Regarding antibiotic combination therapy, lactic acid bacteria can be helpful in minimizing antibiotic dosage and returning the intestinal microbiota back to normal during bacterial infections (Ivanovic et al., 2021). Example: combined with antibiotics against Clostridium difficile infection, the treatment cycle becomes shorter and the probability of recurrence lower. Composite preparations of Chinese herbal polysaccharides and lactic acid bacteria can be used with traditional Chinese medicine to enhance antiviral activity. As vaccine adjuvants, lactic acid bacteria enhance the mucosal immune responses and thus the protective efficacy of vaccines like the foot-and-mouth disease vaccine (Ni et al., 2024). Although murine models included in this review provide useful mechanistic clues, extrapolation of these findings to cattle should be interpreted with caution due to substantial physiological and immunological differences between species. These findings are not directly transferable to bovine clinical practice, and priority should be given to results validated in bovine-specific models. Future research should focus on confirming these pathways directly in the target species.

## 8 Conclusion

The action of lactic acid bacteria is multifactorial in terms of several therapeutic activities concerning the variety of bovine diseases. Firstly, lactic acid bacteria modulate gastrointestinal microbial homeostasis, decrease intestinal pH by producing organic acids like lactic acid, and prevent the growth and proliferation of harmful bacteria thus preserve the health of intestines. Such effects are very useful in curing digestive disorders, such as diarrhoea and indigestion, in cattle. The second reason is that lactic acid bacteria have immunomodulatory properties that activate bovine immunity, increase the responsiveness of the host immunity, increase disease resistance, and also help in preventing and treating infectious diseases due to bacteria and viruses. Besides, lactic acid bacteria generate diverse useful metabolites, i.e., antimicrobial compounds, vitamins, and enzymes, which directly inhibit growth of the pathogens, help to digest and absorb nutrients, and generally enhance the health of the cattle. At the same time, lactic acid bacteria create a biological barrier on the intestinal mucosal surface to prevent the invading pathogens and enhance the intestinal defense mechanism. With all these mechanisms, lactic acid bacteria can be considered as having a significant role in bovine digestive and infectious disease treatment. Lactic acid bacteria have shown great potential as a therapeutic approach to bovine diseases and are recognized as safe with high safety margins. As common probiotics found in nature, lactic acid bacteria are non-toxic to cattle, do not cause antibiotic resistance and do not leave behind any residual effects in animals. The use of lactic acid bacteria has proven to alleviate various bovine diseases, enhanced production performance, increased feed utilization, and decreased morbidity and mortality, and therefore economically beneficial to the livestock sector. Most significantly, lactic acid bacteria are clean organisms and fit in with the contemporary green, eco-friendly, and sustainable breeding principles. In comparison with traditional chemical drugs, the use of lactic acid bacteria minimizes environmental pollution, guarantees the safety and quality of the livestock products, and facilitates the sound and sustainable development of animal farming.
